# Supplementary material for: Social Determinants of the Transition in Food Consumption in Paraíba, Brazil, Between 2008 and 2018
Source: Nutrients. 2025 Aug 4;17(15):2550. doi: 10.3390/nu17152550 (PMC12348789; doi:10.3390/nu17152550)
Supplement: Supplementary file 1 [file nutrients-17-02550-s001.zip › nutrients-3716172-supplementary.pdf]

Figure S1. Flowchart of sample selection.

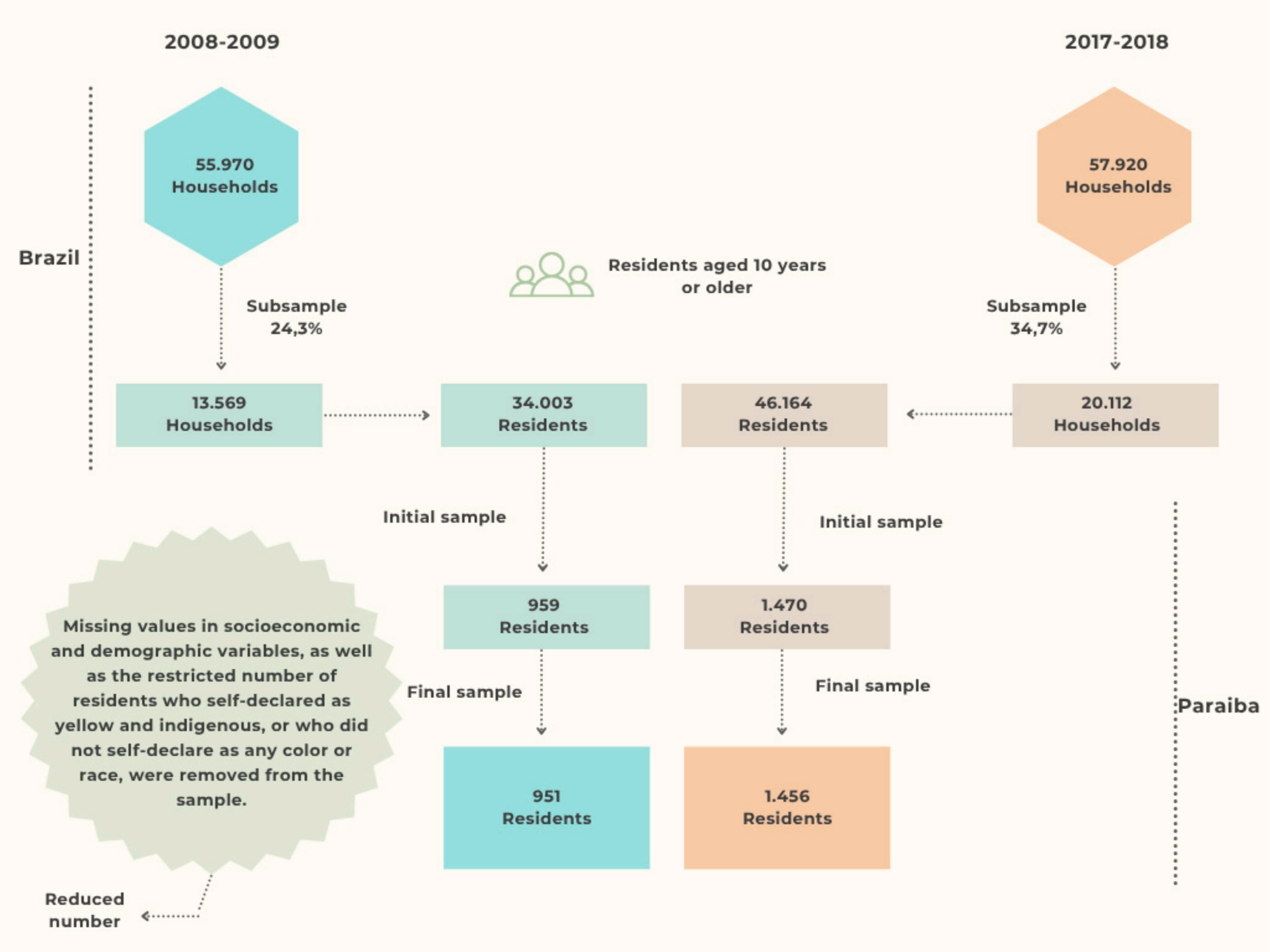

Table S1. Mean caloric intake of food subgroups consumed by the population aged 10 years or older. Data from the State of Paraíba in the POF 2008–2009 (n = 951) and 2017–2018 (n = 1,456).

POF Edition			
2008-2009		2017-2018	
**Food Subgroups**	**Mean Calories**	**Food Subgroups**	**Mean Calories**
Polished/white rice	165.1	Polished/white rice	151
Beef	154	French rolls	145
French rolls	144.1	Sugars	128.4
Beans	132	Poultry	117.6
Oils/olive oil	90.4	Beef	116.8
Poultry	84.9	Beans	114.4
Other cereals	75	Oils/olive oil	102.6
Milk	51.2	Other cereals	67.4
Fruit	49	Pasta	62.2
Savory biscuits/crackers	47.8	Roots and tubers	59
Pasta	42.7	Savory biscuits/crackers	49.5
Sweet biscuits	41	Fruit	45.9
Dried/salted/smoked meats	40.1	Processed cheese	36.3
Roots and tubers	33.2	Margarine	34.5
Processed cheese	32.9	Milk	33.9
Eggs	32.6	Eggs	32.9

Table S1. Cont.

POF Edition			
2008-2009		2017-2018	
**Food Subgroups**	**Mean Calories**	**Food Subgroups**	**Mean Calories**
Fish	30.9	Sweet biscuits	32.4
Sugars	27.7	Processed meats/sausages	28.2
Vegetables	26.8	Pork	27.7
Butter	21.7	Dried/salted/smoked meats	27
Natural juice	21.4	Ultra-processed bread	27
Cassava flour	20.3	Other culinary ingredients	26.2
Margarine	18.5	Cassava flour	23.3
Soda	17.4	Vegetables	22.9
Coffee and tea	16.2	Natural juice	21.8
Yogurts and dairy drinks	16	Butter	17.9
Other culinary ingredients	12.5	Fermented	14.8
Chocolate, candy, etc.	11.9	Soda	13.9
Processed meats/sausages	10.8	Fish	13.2
Ready-made pasta dishes	10.4	Artificial juice	12.6
Pork	9.3	Yogurts and dairy drinks	10.9
Wheat flour	9.2	Coffee and tea	10.4
Ultra-processed bread	9.2	Wheat flour	10.3
Fruit preserves	8.2	Salty snacks	10.1
Ice cream	7.6	Pizza	9.2
Pizza	7.3	Other flours	8.5
Sandwiches	7.3	Sandwiches	8.1
Salty snacks	7.1	Instant noodles	7.6
Artificial juice	7.1	Other sweets	7.1
Preserved/dried/salted fish	6.3	Variety meats	6.7
Brown rice	6.2	Savory pastries	6.6
Fast food	6.1	Fruit preserves	5.8
Other sweets	5.6	Distilled spirits	5.5
Other legumes	5.2	Fast food	5.4
Variety meats	4.9	Other meats	4.4
Other meats	4.7	Ice cream	4.4
Savory pastries	4.4	Ready-made pasta dishes	4.1
Distilled spirits	4.2	Chocolate, candy, etc.	3.9
Natural yogurt	4	Sweet bread, cakes, pastries	3.7
Other flours	3.8	Industrialized sauces	3.6
Instant noodles	3.2	Bacon	3.5
Breakfast cereals	2	Brown rice	2.8
Sweet bread, cakes, pastries	2	Other legumes	2.4
Bacon	1.8	Natural yogurt	2.2
Seafood and others	1.4	Nuts and seeds (incl. peanuts)	1.9
Industrialized sauces	0.7	Preserved/dried/salted fish	1.8
Other non-alcoholic beverages	0.4	Breakfast cereals	1.7
Tomato sauce	0.1	Cream	1.6
		Ultra-processed cheese	1.3
		Preserved legumes	0.5
		Formulas and supplements	0.5
		Preserved vegetables	0.3
